# Delayed Recognition and Stroke-Predominant Presentation in Down Syndrome–Associated Moyamoya Syndrome

**DOI:** 10.1161/STROKEAHA.126.056969

**Published:** 2026-07-24

**Authors:** Jonathan D. Santoro, Mackenzie Silverman, Anthony C. Wang, Deepti Nagesh, Eugenia Ho, Maeve C. Lucas, Samuel T. Otey, Mariam M. Yousuf, Sarah Lee, Elizabeth W. Mayne, Brian G. Skotko, Gary K. Steinberg, Michael S. Rafii

**Affiliations:** Division of Neurology, Department of Pediatrics, Children’s Hospital Los Angeles, CA (J.D.S., M.S., D.N., E.H., M.C.L., S.T.O., M.M.Y.).; Department of Neurology, Keck School of Medicine of the University of Southern California, Los Angeles (J.D.S., D.N., E.H., M.S.R.).; Department of Neurosurgery, University of California at Los Angeles (A.C.W.).; Department of Neurology (S.L., E.W.M.), Stanford University School of Medicine 10624 , CA.; Department of Neurosurgery (G.K.S.), Stanford University School of Medicine 10624 , CA.; Department of Pediatrics, Harvard Medical School, Boston, MA (B.G.S.).; Down Syndrome Program, Division of Medical Genetics and Metabolism, Department of Pediatrics, Massachusetts General Hospital 2348 , Boston (B.G.S.).; Alzheimer’s Therapeutic Research Institute, Keck School of Medicine of the University of Southern California, San Diego (M.S.R.).

**Keywords:** blood pressure, Down syndrome, moyamoya disease, odds ratio, stroke

## Abstract

**BACKGROUND:**

Children with Down syndrome (DS) are at high risk for moyamoya syndrome (MMS) and ischemic stroke, yet early detection strategies remain poorly defined despite the condition being surgically treatable.

**METHODS:**

We conducted a multicenter retrospective cohort study comparing children with Down syndrome–associated moyamoya syndrome (DS-MMS) and MMS without DS (MMS). The primary outcome was stroke as the primary presenting symptom. Secondary outcomes included diagnostic delays, angiographic features, prediagnostic systolic blood pressure percentiles, and 1-year neurological outcomes. Multivariable models adjusted for demographic and access-related covariates.

**RESULTS:**

In total, 271 patients were identified; 198 (73.1%) met inclusion criteria and comprised the analytic cohort. Among 198 patients (77 DS-MMS; 121 MMS), DS-MMS mean age was 9.5±5.2 years (50.6% female patients), and MMS mean age was 7.4±3.0 years (54.5% female patients). Stroke at presentation was more common in DS-MMS (71.4% versus 20.7%; absolute difference, 50.8%), corresponding to an adjusted odds ratio of 14.50 ([95% CI, 6.65–31.60]; *P*<0.001). Children with DS-MMS experienced longer delays from symptom onset to presentation and from presentation to diagnostic confirmation (both *P*<0.001). Posterior circulation involvement was more frequent in DS-MMS (adjusted odds ratio, 2.18 [95% CI, 1.09–4.37]; *P*=0.03), whereas angiographic severity was similar between groups. In the prediagnostic period, DS-MMS demonstrated a progressive rise in systolic blood pressure percentiles, exceeding the MMS cohort by 6 months (*P*<0.001). At 1 year, DS-MMS was associated with greater disability (adjusted odds ratio, 2.58 [95% CI, 1.45–4.57]; *P*<0.001) and spasticity (adjusted odds ratio, 6.33 [95% CI, 3.12–12.83]; *P*<0.001).

**CONCLUSIONS:**

DS-MMS represents a high-risk cerebrovascular phenotype characterized by delayed recognition and a markedly increased likelihood of stroke at presentation. Rising blood pressure percentiles preceding diagnosis may serve as an early physiological signal. These findings support targeted early detection strategies and a lower threshold for vascular imaging in symptomatic patients.

Children with Down syndrome (DS) represent one of the highest-risk pediatric populations for moyamoya syndrome (MMS) and ischemic stroke, yet strategies for early detection remain poorly defined. Because MMS is a surgically treatable cause of stroke, delays in recognition represent a potentially modifiable source of neurological morbidity.^1,2^ MMS is characterized by progressive stenosis or occlusion of the terminal internal carotid arteries and their proximal branches, with development of fragile collateral networks.^3^ In children, the disease most commonly manifests with ischemic events, transient ischemic attacks or infarction, although hemorrhage can occur.^4,5^ As pediatric cerebrovascular injury can disrupt development during critical windows, the consequences of delayed recognition are substantial, with long-term impacts on motor function, cognition, behavior, school participation, and family quality of life.^6–8^

The association between DS and moyamoya syndrome (DS-MMS) is well recognized, but the clinical presentation can be heterogeneous and may be difficult to detect early.^1,2^ Symptoms may include episodic weakness, speech disturbance, seizures, headaches, or more subtle changes in gait, attention, or behavior that can be challenging to distinguish from baseline neurodevelopmental variability or common DS comorbidities.^1,2,9^ Children with DS also frequently have co-occurring medical conditions, such as congenital heart disease, sleep-disordered breathing, and thyroid dysfunction, which can complicate neurological assessment or influence cerebrovascular physiology.^10^ As a result, timely diagnosis often requires a high index of suspicion and careful attention to transient or atypical neurological symptoms. MMS is a surgically correctable condition, making the need for early diagnosis even more critical as it may obviate the development of neurological disability.^8^

Despite the clinical relevance of DS-MMS, important gaps remain in how best to identify at-risk children, standardize evaluation pathways, and optimize multidisciplinary follow-up tailored to DS-specific needs. A clearer understanding of DS-MMS epidemiology, presenting symptom patterns, comorbid profiles, and posttreatment functional trajectories is needed to inform targeted screening approaches, expedite diagnosis, and reduce preventable neurological injury.

This study sought to evaluate differences in the time to evaluation and diagnosis of cerebrovascular disease between children with DS-MMS and MMS without DS in a multicenter retrospective cohort. The authors sought to evaluate clinical and demographic factors that may influence potential delays in evaluation and diagnosis. Finally, the authors sought to evaluate if delays in evaluation and diagnosis affected long-term neurological outcomes in both cohorts.

## Methods

### Ethics Approval and Data Availability

This study was approved by the Children’s Hospital Los Angeles/University of Southern California Institutional Review Board (No. 20-00729). As the nature of this study was retrospective in nature, consent and assent were waived. The data that support the findings of this study are available from the corresponding author on reasonable request and institutional review board approval.

### Patient Selection

Cases were identified retrospectively from a prospectively enrolled, multicenter moyamoya disease research database after obtaining institutional review board approval. Cases in the DS-MMS cohort fulfilled the following criteria: (1) diagnosis of DS confirmed by karyotype, (2) a diagnosis of MMS based on published angiographic criteria,^11^ and (3) clinical evaluation between January 1, 1998 and September 1, 2025. Patient demographics, medical comorbidities, and clinical presentation of MMS were collected via chart review. Patients were excluded if they had clinically significant thyroid disease, heart disease, renal artery stenosis, or untreated obstructive sleep apnea. Patients were excluded if these findings were clinically significant, suboptimally treated, or untreated (Supplemental Methods).^9^ All available vascular imaging, including computed tomography angiography, magnetic resonance angiography, and catheter angiography, were reviewed to confirm MMS diagnosis. Imaging review was performed by a centralized board-certified pediatric neuroradiologist, and Suzuki stage and posterior circulation involvement were assigned retrospectively using published criteria.^11^

The control group of individuals with MMS without DS was identified through both prospective institutional (single center) databases and retrospectively by searching electronic medical records for patients with an established *International Classification of Diseases, Ninth Revision* and *International Classification of Diseases, Tenth Revision* code for MMS (437.5 and I67.5, respectively) from January 1, 1998, and September 1, 2025. Individuals in the MMS cohort could have any genetic diagnosis except for DS. Identical exclusion criteria were applied to this cohort, and confirmation of MMS diagnosis was identical to the DS-MMS group.

### Operational Definitions and Classifications

- Clinical presentation: Defined as the first evaluation for a neurological sign or symptom which was eventually attributed to MMS. This could be in any clinical environment (eg, emergency department, outpatient primary care clinic).
- Confirmation of diagnosis: Defined as the first dedicated vascular imaging that confirmed the presence of moyamoya vascular findings. This could include either computed tomography angiography, magnetic resonance angiography, or angiography.
- Spasticity at 1 year: Defined as having increased tone on neurological examination in ≥1 extremities 1 year after diagnosis.
- Intellectual capabilities worsened at 1 year: Defined as definitive neuropsychological testing demonstrating cognitive decline, subjective report by a clinician of cognitive decline in medical documentation, or documentation of changes to educational program at school (eg, new or revised individualized educational plan) 1 year after diagnosis. Prestroke disability was not adjusted for in the primary modified Rankin Scale (mRS) score models because a standardized prestroke mRS score or comparable functional measure was not consistently available across sites.

### Follow-Up and Missing Data

One-year outcomes were abstracted from routinely collected clinical documentation, including neurology, neurosurgery, rehabilitation, and primary care follow-up notes. Outcomes were assigned using the clinical encounter closest to 12 months after diagnosis within a prespecified window of 9 to 15 months. If no documentation was available within this window, the outcome was treated as missing for that analysis.

### Blood Pressure Evaluation

Consistent with previously published literature by the authors,^9^ blood pressure (BP) data were extracted from outpatient clinical notes and encounters, which were nonemergent in nature. Urgent visits for conditions not expected to be associated with changes in BP, such as conjunctivitis, were included. All cases had 1 BP measurement during each of the categorical time intervals (24, 18, 12, and 6 months) before diagnosis of MMS. A range of ±2 months was applied to these date frames. To allow comparison across ages, BP measurements were converted to percentiles for age and height by using standardized data from the National Heart, Lung, and Blood Institute.^12^ BP elevation was defined as an increase of ≥25 systolic BP percentile points from the 24-month value at the 18-, 12-, or 6-month assessments. For the 24-month assessment, BP elevation was defined relative to the earliest available prediagnostic outpatient BP measurement preceding that window.

### Statistical Analysis

Continuous variables were summarized as median (interquartile range [IQR]) and compared using Wilcoxon rank-sum tests; categorical variables were reported as counts (percentages) and compared using Fisher exact or χ^2^ tests as appropriate. Multivariable regression models assessed the association between DS status and outcomes, adjusting for sex, age, race, Hispanic ethnicity, primary language, and insurance type. To account for skewness, delay outcomes were analyzed on a log(1+days) scale. Binary outcomes (eg, stroke at presentation, spasticity) were evaluated using logistic regression (adjusted odds ratios [aORs]), whereas ordinal outcomes (mRS score and Suzuki stage) utilized proportional odds models.

To account for secular changes, models were sensitivity-adjusted for diagnosis era (1998–2006, 2007–2014, and 2015–2025). The predictive value of BP elevation for 1-year outcomes was evaluated using adjusted logistic models. Median differences and 95% CIs for continuous outcomes were estimated via bootstrap resampling. Rare outcomes with sparse event counts resulting in unstable estimates were considered not estimable.

To evaluate the predictive value of BP on outcomes, we examined elevated BP at 6, 12, 18, and 24 months (yes/no) as a prespecified predictor of 1-year outcomes using unadjusted and adjusted logistic models. Adjusted models included DS status and the same covariates listed above. For rare outcomes with sparse event counts resulting in unstable model estimates, effect sizes were considered not estimable.

Missing data were handled using complete-case analysis for each outcome-specific model. No imputation was performed. Denominators are reported when they vary across outcomes, and patients with missing exposure, outcome, or covariate data were excluded only from the relevant analysis. The study is reported in accordance with STROBE guidelines (Strengthening the Reporting of Observational Studies in Epidemiology) for observational studies. All statistical tests were 2-sided, with *P*<0.05 considered statistically significant.

## Results

### Study Population

In total, 271 individuals were identified for inclusion, although only 211 (77.8%) had complete data available. Data for patients excluded due to incomplete data are provided in Table S1. After secondary review of complete data sets, 13 (6.1%) individuals were excluded for having clinically significant, untreated, or suboptimally treated hypothyroidism (4/13 DS-MMS, 0/13 MMS), cardiac disease (4/13 DS-MMS, 2/13 MMS), and obstructive sleep apnea (2/13 DS-MMS, 1/13 MMS; Table S2). Of eligible individuals, 77 (38.9%) had DS-MMS and 121 had MMS without DS. Age at presentation did not differ significantly between groups (median, 7.6 versus 7.3 years). Demographic data are presented in Table 1. Of individuals in the MMS cohort, 63 (52.1%) were cryptogenic, 35 (28.9%) had sickle cell disease, 11 (9.1%) had neurofibromatosis type 1, 11 (9.1%) had prior radiotherapy, and 1 (0.8%) had progeria. Of this cohort, only 9 patients (all with sickle cell disease) were receiving routine transcranial Doppler ultrasound screening. When dichotomized as 2015 to 2025 versus earlier years at the time of presentation, the odds of being diagnosed in the later era were similar for DS-MMS versus MMS (OR, 0.86 [95% CI, 0.48–1.52]; *P*=0.66).

**Table 1.**
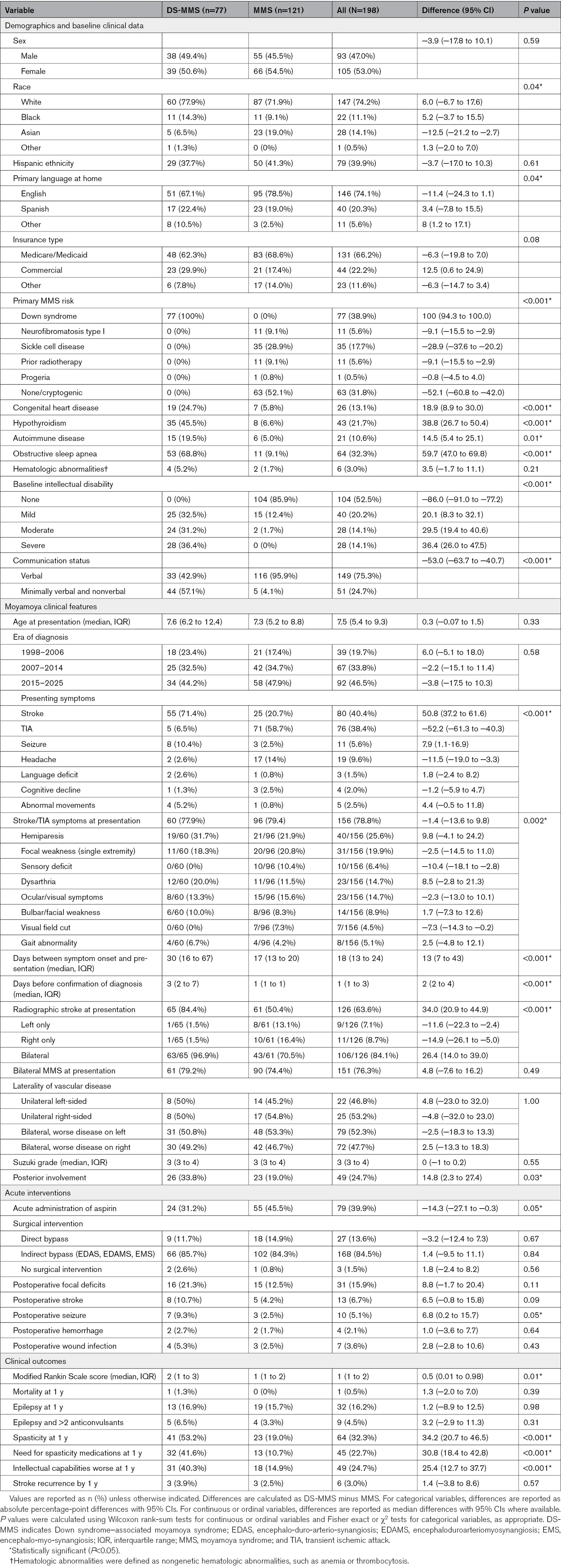
Clinical and Demographic Data for Individuals With DS-MMS Versus MMS

### Delays in Care

**Figure 1.**
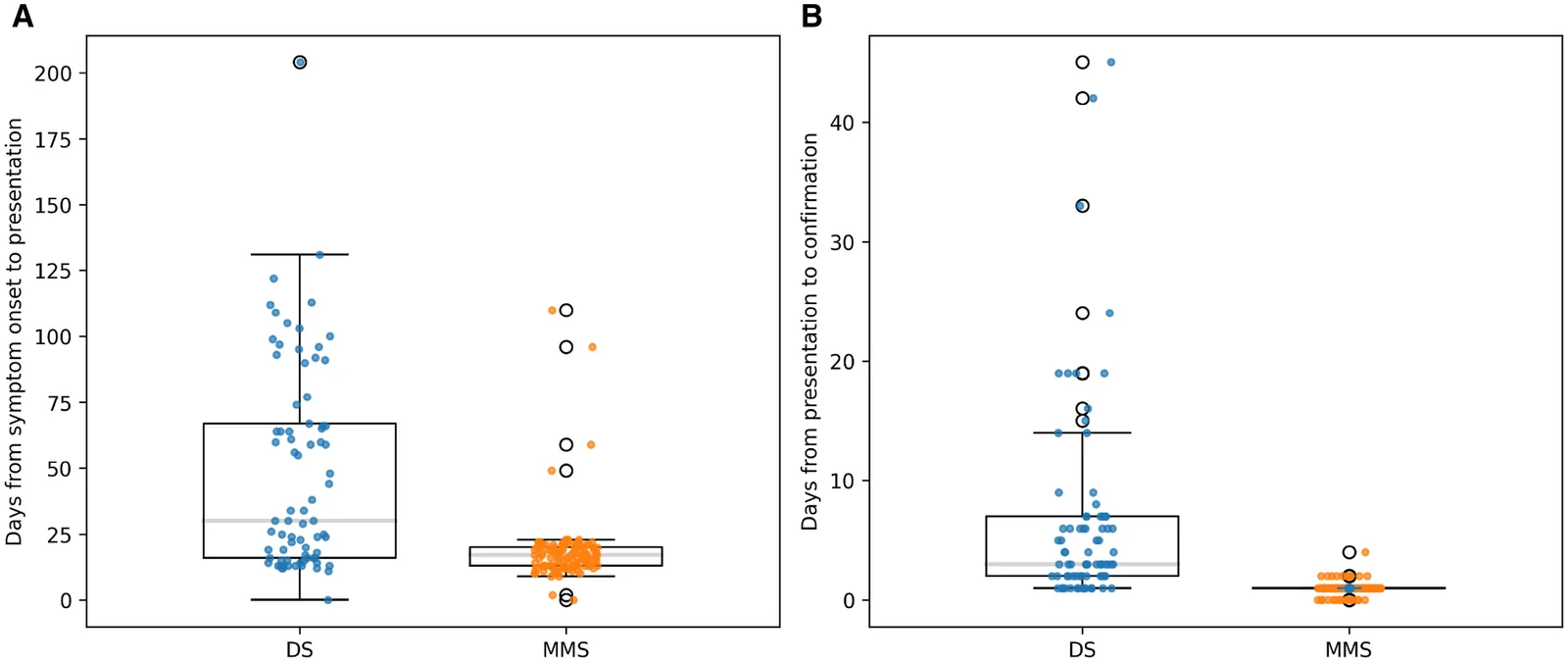
**Diagnostic delays from symptom onset to presentation and from presentation to diagnostic confirmation in children with Down syndrome–associated moyamoya syndrome (DS-MMS) compared with moyamoya syndrome without Down syndrome (MMS). A**, Number of days from symptom onset to first clinical presentation. **B**, Number of days from clinical presentation to diagnostic confirmation by vascular imaging. Individual data points represent patients, with box plots displaying the median, interquartile range, and whiskers extending to 1.5× the interquartile range; outliers are shown as individual points. Comparisons between groups were performed using the Wilcoxon rank-sum test due to non-normal distributions. Median differences and corresponding 95% CIs were estimated using Hodges-Lehmann estimators with bootstrap resampling. In multivariable analyses using log(1+days)–transformed outcomes, Down syndrome status was independently associated with longer delays in both the symptom onset-to-presentation interval (adjusted ratio, 1.97 [95% CI, 1.59–2.43]; *P*<0.001) and the presentation-to-confirmation interval (adjusted ratio, 2.78 [95% CI, 2.36–3.26]; *P*<0.001). The experimental unit is the individual patient.

Children with DS-MMS experienced substantially longer delays in care (Table 1; Figure 1). The median time from symptom onset to presentation was 30 days (IQR, 16–67) in DS-MMS versus 17 days (IQR, 13–20) in MMS (*P*<0.001). In addition, the time from presentation to diagnostic confirmation was 3 days (IQR, 2–7) in DS-MMS versus 1 day (IQR, 1–1) in MMS (*P*<0.001).

In adjusted analyses (Table 2), DS-MMS remained independently associated with longer delays, including a 2.23-fold longer total delay (adjusted ratio, 2.23 [95% CI, 1.85–2.70]; *P*<0.001), a 1.97-fold longer symptom onset-to-presentation interval (adjusted ratio, 1.97 [95% CI, 1.59–2.43]; *P*<0.001), and a 2.78-fold longer presentation-to-confirmation interval (adjusted ratio, 2.78 [95% CI, 2.36–3.26]; *P*<0.001), after accounting for sex, age at presentation, race, ethnicity, primary language, and insurance type. Era of diagnosis was not associated with delays in presentation (*P*=0.81) or confirmation (*P*=0.63) for individuals with DS-MMS but was associated with shorter time to presentation and confirmation in the MMS cohort for individuals with sickle cell disease ([95% CI, 1.04–5.12]; *P*=0.01 and [95% CI, 2.11–3.47]; *P*<0.001, respectively) diagnosed after 2006.

**Table 2.**
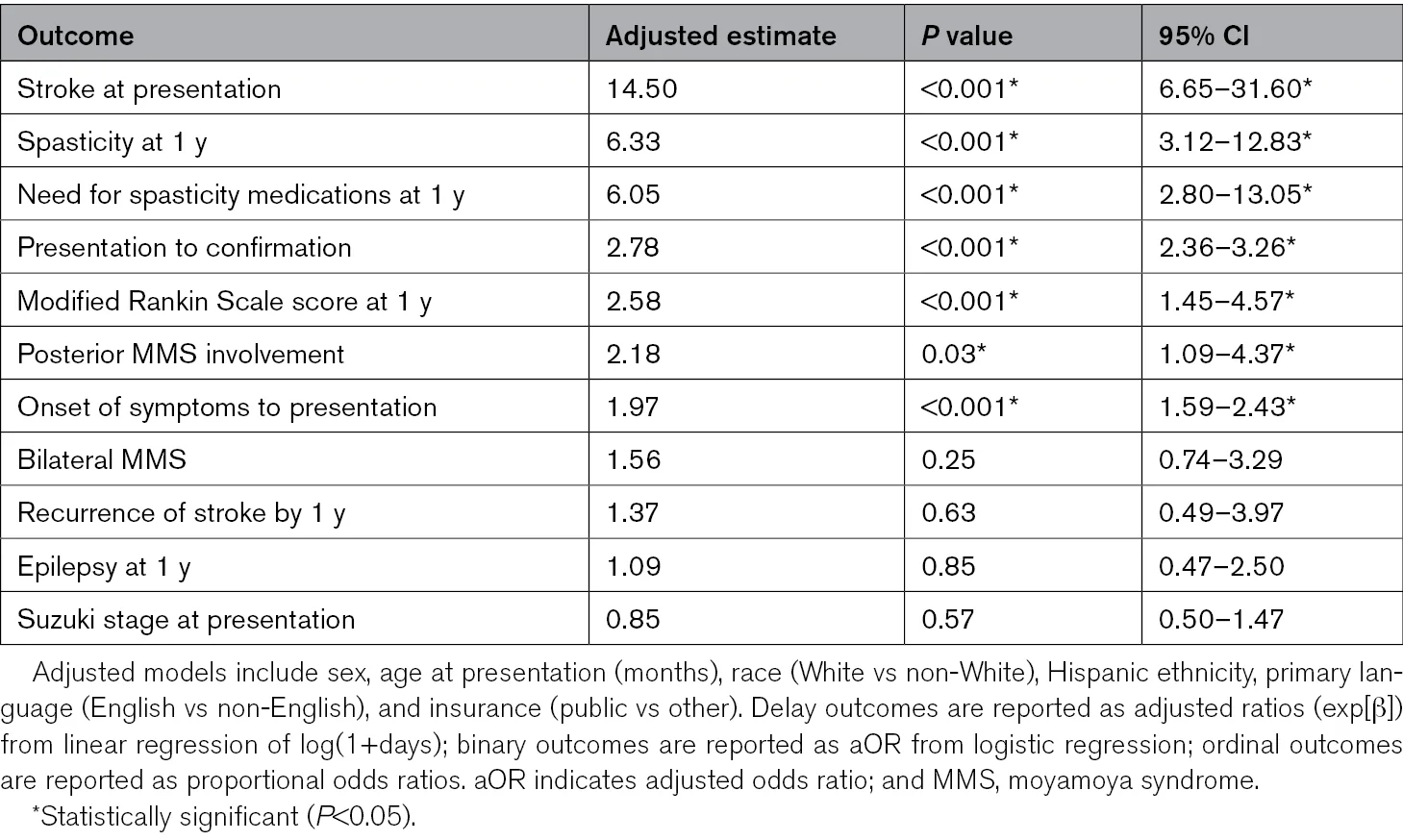
Adjusted Estimate of Down Syndrome Status With Delays in Care, Presentation Phenotype, Angiographic Features, and 1-Year Outcomes

Within the DS-MMS cohort, baseline neurodevelopmental status significantly influenced the timeline of care. Increasing severity of baseline intellectual disability (ID) was stepwise associated with longer median delays from symptom onset to clinical presentation (mild: 16.0 days [95% CI, 12.2–19.8]; moderate: 42.5 days [95% CI, 29.9–55.1]; severe: 82.0 days [95% CI, 59.9–104.1]; *P*<0.001) as well as delays from presentation to diagnostic confirmation (*P*=0.0016). Interestingly, verbal patients experienced significantly longer delays from symptom onset to presentation compared with minimally verbal or nonverbal patients (61.0 days [95% CI, 41.6–80.4] versus 22.5 days [95% CI, 12.1–32.9]; *P*=0.002). A parallel neurodevelopmental stratification analysis was not performed in the MMS cohort because baseline ID and communication differences were uncommon and sparsely distributed in this group, limiting the ability to generate stable estimates across neurodevelopmental strata.

### Presentation Phenotype and Cerebrovascular Features

**Figure 2.**
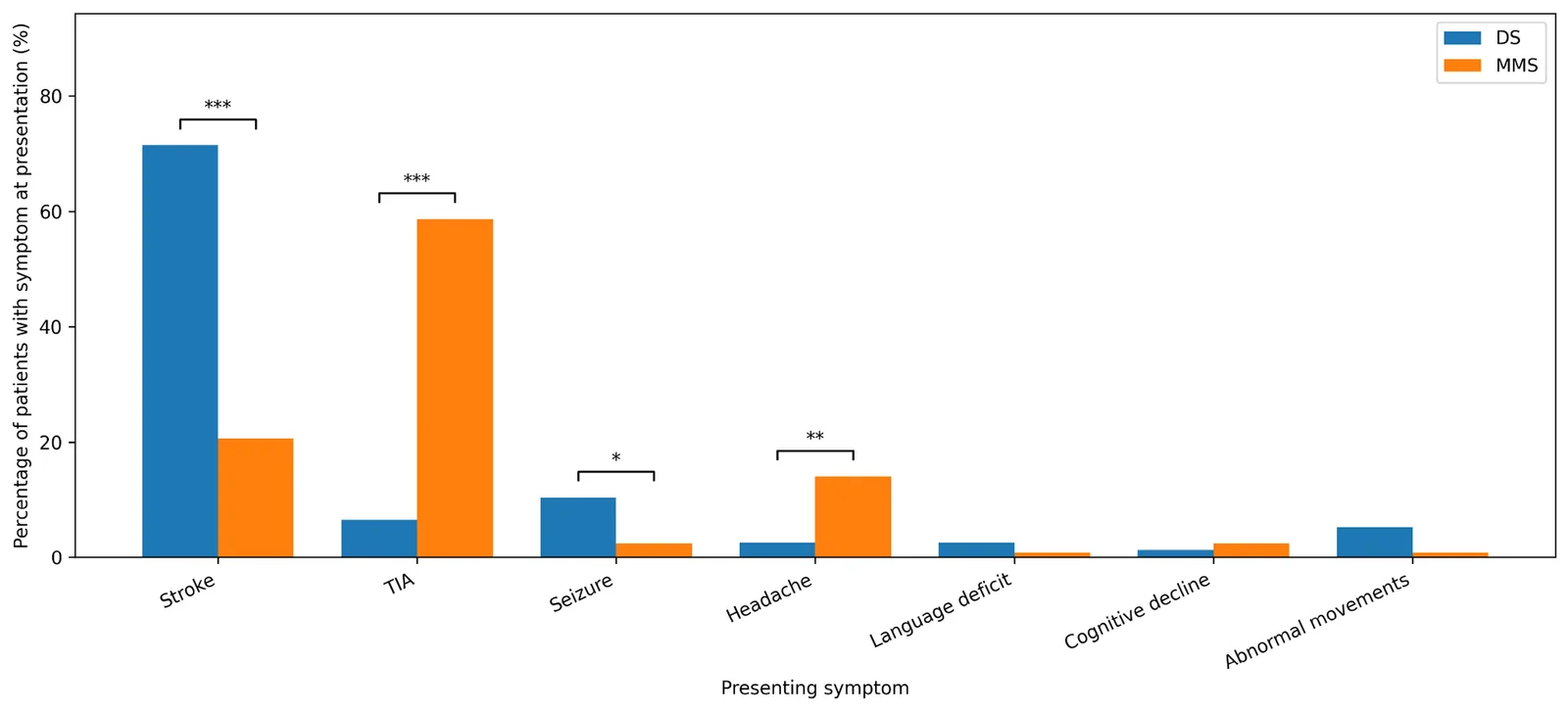
**Presenting neurological symptoms in children with Down syndrome–associated moyamoya syndrome (DS-MMS) compared with moyamoya syndrome (MMS).** Bars represent the percentage of patients presenting with each symptom category, including stroke, transient ischemic attack (TIA), seizure, headache, language deficit, cognitive decline, and abnormal movements. The experimental unit is the individual patient. Group comparisons were performed using χ^2^ tests or Fisher exact tests as appropriate. Stroke was significantly more common in DS-MMS compared with MMS (71.4% vs 20.7%), corresponding to an unadjusted odds ratio of 9.60 ([95% CI, 4.95–18.61]; *P*<0.001). Asterisks denote statistical significance (*P<0.05; **P<0.01; ***P<0.001), with exact *P* values and additional effect estimates provided in Table 1.

Clinical presentation differed markedly by group (Table 1; Figure 2). The absolute risk difference for stroke at presentation was 50.8% (DS-MMS [55/77 (71.4%)] versus MMS [25/121 (20.7%)]), corresponding to an unadjusted OR of 9.60 ([95% CI, 4.95–18.61]; *P*<0.001) and number-needed-to-miss of ≈2. After adjustment, DS status remained strongly associated with stroke as the presenting symptom (aOR, 14.50 [95% CI, 6.65–31.60]; *P*<0.001; Table 2). When the presence of radiographic stroke at initial evaluation was analyzed separately from the primary presenting symptom, stroke was also more common in DS-MMS than MMS (65/77 [84.4%] versus 61/121 [50.4%]; OR, 5.33 [95% CI, 2.62–10.85]; *P*<0.001). Among patients with stroke present at initial evaluation, bilateral stroke was more frequent in DS-MMS than MMS (63/65 [96.9%] versus 43/61 [70.5%]), whereas isolated left- or right-sided stroke was less common (overall laterality distribution *P*<0.001). In covariate models, Hispanic ethnicity was associated with a longer presentation-to-confirmation interval (adjusted ratio 1.28 [95% CI, 1.08–1.51]; *P*=0.01).

Suzuki staging was similar between cohorts with a median grade of 3 (IQR, 3–4) in both groups (Table 1). Suzuki stage was not associated with DS status in adjusted modeling (ordinal OR, 0.85 [95% CI, 0.50–1.47]; *P*=0.57; Table 2). Laterality did not differ between cohorts (*P*=0.54). Among unilateral cases, right- and left-sided disease were similarly distributed. Among bilateral cases, the more severely affected hemisphere was also balanced between right and left across cohorts (Table 1).

Posterior circulation involvement was more frequent in individuals with DS-MMS (unadjusted OR, 2.17 [95% CI, 1.13–4.18]; *P*=0.03), and this association persisted after adjustment (aOR, 2.18 [95% CI, 1.09–4.37]; *P*=0.03; Table 2). Because posterior circulation involvement was more common in DS-MMS, sensitivity analyses additionally adjusting for posterior involvement were performed. DS-MMS remained independently associated with longer symptom onset-to-presentation delay (adjusted ratio, 2.01 [95% CI, 1.60–2.52]; *P*<0.001) and longer presentation-to-confirmation delay (adjusted ratio, 2.66 [95% CI, 2.24–3.16]; *P*<0.001). Posterior involvement itself was not independently associated with either delay outcome.

### BP Abnormalities Preceding Presentation

**Figure 3.**
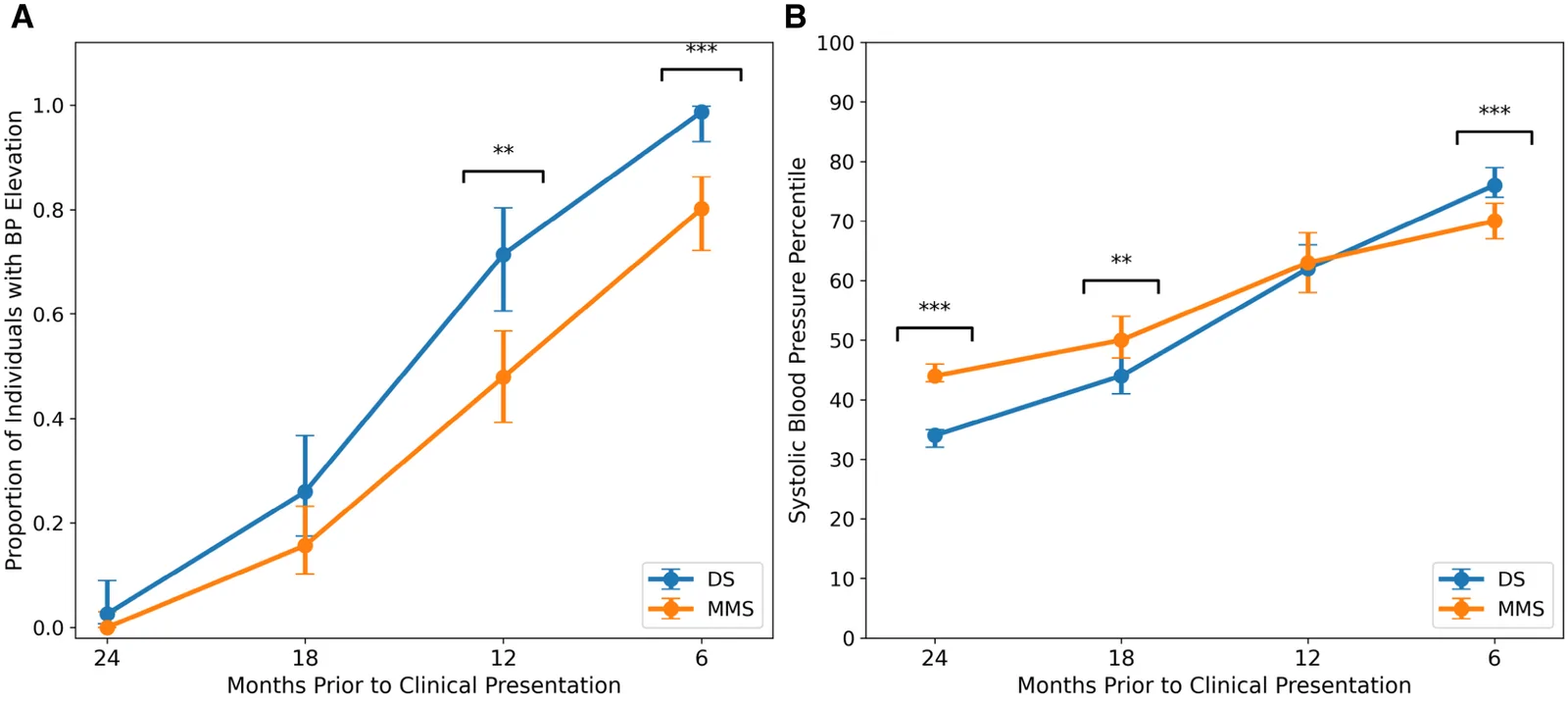
**Longitudinal systolic blood pressure (BP) patterns preceding diagnosis of moyamoya syndrome (MMS). A**, Proportion of patients with elevated systolic BP at 24, 18, 12, and 6 months before diagnosis, defined as an increase of ≥25 percentile points compared with prior measurements. **B**, Systolic BP percentiles over the same time intervals. Time is displayed as months before diagnosis. In **A**, proportions are shown with 95% CIs, and comparisons between groups at each time point were performed using Fisher exact tests. In **B**, points represent mean systolic BP percentiles with SE bars, and comparisons between groups were performed using Welch *t* tests. Differences in the proportion of elevated BP were not significant at 24 months (*P*=0.15) or 18 months (*P*=0.10), but were significant at 12 months (*P*=0.001) and 6 months (*P*<0.001). Systolic BP percentiles were lower in Down syndrome–associated moyamoya syndrome (DS-MMS) at 24 months (*P*<0.001) and 18 months (*P*=0.01), not different at 12 months (*P*=0.73), and higher in DS-MMS at 6 months (*P*<0.001), demonstrating a dynamic trajectory preceding diagnosis. The experimental unit is the individual patient. The *x* axis is displayed in reverse chronological order (24–6 months prior). Significance annotations indicate between-group differences at each time point (*P<0.05, **P<0.01, and ***P<0.001).

Premorbid BP data were available for all 198 patients in the analytic cohort. Each patient contributed 1 BP measurement at each of 4 prespecified intervals before MMS diagnosis: 24, 18, 12, and 6 months, yielding 792 total BP readings. In longitudinal prepresentation assessments, individuals with DS-MMS demonstrated a higher prevalence of BP elevation than the MMS cohort at later prediagnostic time points (Table 3; Figure 3A). The proportion with BP elevation did not differ at 24 (*P*=0.15) and 18 months (*P*=0.10) but was significantly higher in DS at 12 months (*P*=0.001) and 6 months (*P*<0.001). Consistent with these findings, systolic BP percentiles differed by group over time (Figure 3B), with the DS-MMS cohort having lower percentiles at 24 months (*P*<0.001) and 18 months (*P*=0.003), no difference at 12 months (*P*=0.73), and higher percentiles at 6 months (*P*<0.001). Bilateral disease was associated with a higher systolic BP percentile at 12 months before diagnosis (mean difference, 7.6 percentile points [95% CI, 1.1–14.1]; *P*=0.02), although categorical BP elevation at 12 months showed only a nonsignificant trend after adjustment (OR, 1.96 [95% CI, 0.95–4.05]; *P*=0.07).

**Table 3.**
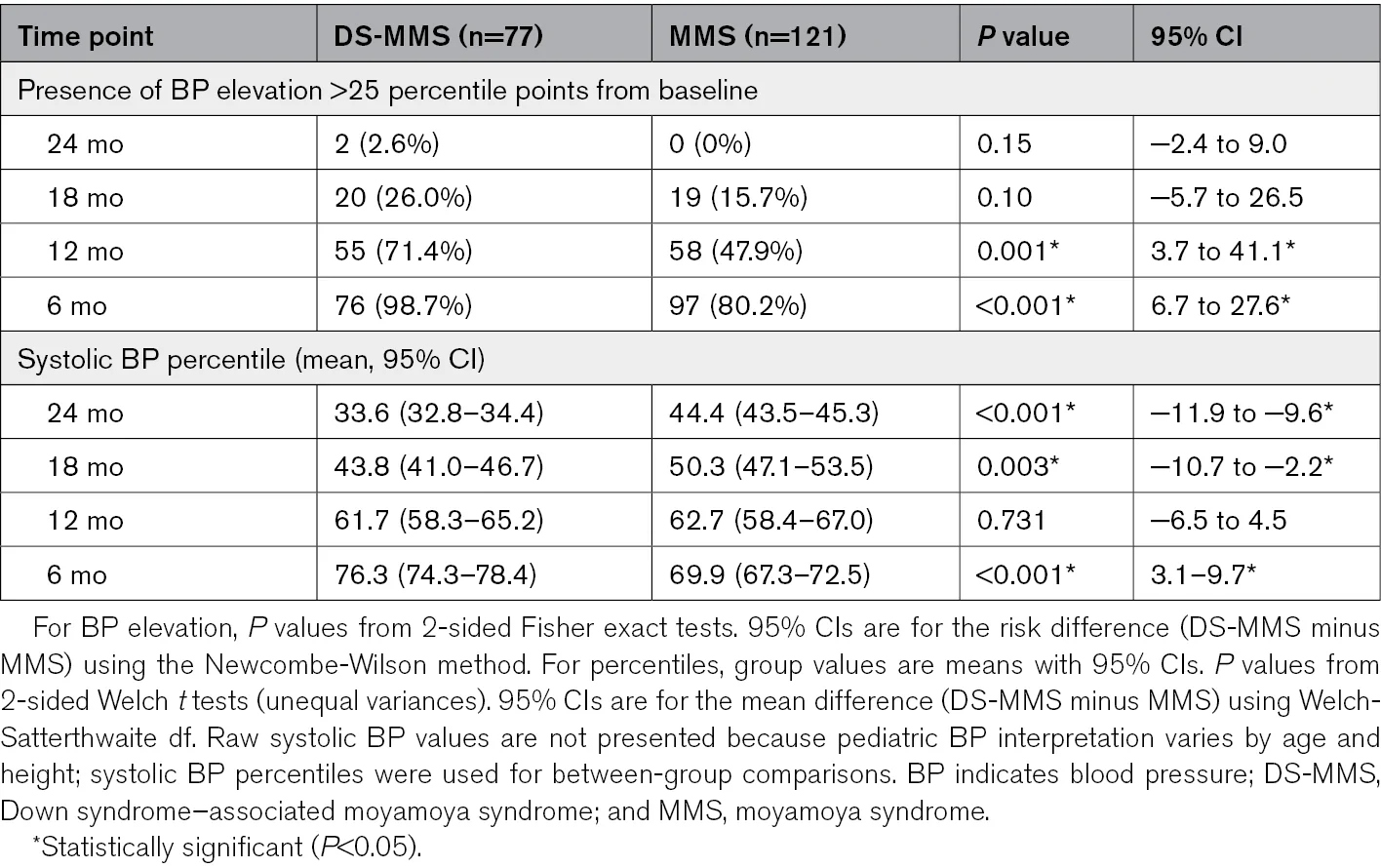
Age- and Height-Adjusted Systolic BP Percentiles Before Diagnosis by Cohort

### Acute Interventions

Most patients underwent indirect revascularization (DS-MMS: 66/77 [85.7%]; MMS: 102/121 [84.3%]), with no significant difference in the rates of direct bypass, indirect bypass, or no surgical intervention between cohorts. Acute utilization of aspirin was numerically lower in DS-MMS (31.2% versus 45.5%; *P*=0.05). After adjustment, DS-MMS was not independently associated with aspirin administration (aOR, 0.67 [95% CI, 0.35–1.27]; *P*=0.22) or direct bypass surgery (aOR, 0.79 [95% CI, 0.32–1.94]; *P*=0.61). As nearly all patients underwent surgical revascularization, adjusted models comparing any surgical intervention versus no surgical intervention were unstable and are not reported. Postoperative complications were uncommon; postoperative seizures were numerically more frequent in DS-MMS (9.3% versus 2.5%; *P*=0.05), whereas postoperative stroke, hemorrhage, wound infection, and recurrent stroke at 1 year did not differ significantly (Table 1).

### One-Year Outcomes

Disability measured by mRS score was higher in DS-MMS (median 2 [IQR 1–3]) compared with MMS (median, 1 [IQR 1–2]; *P*=0.01; Table 1). In adjusted ordinal modeling, DS status was associated with worse disability category (ordinal OR, 2.58 [95% CI, 1.45–4.57]; *P*<0.001; Table 2). Spasticity (unadjusted OR, 4.85 [95% CI, 2.56–9.18]; *P*<0.001) and the need for spasticity medications (unadjusted OR, 5.91 [95% CI, 2.84–12.29]; *P*<0.001) were more common in individuals with DS (Table 1). These associations persisted after adjustment (spasticity: aOR, 6.33 [95% CI, 3.12–12.83]; *P*<0.001; spasticity medications: aOR, 6.05 [95% CI, 2.80–13.05]; *P*<0.001; Table 2). Recurrent stroke and mortality within 1 year were uncommon in both groups (Table 1). Epilepsy was similar between groups (*P*=0.98) and remained nonsignificant after adjustment (aOR, 1.09 [95% CI, 0.47–2.50]; *P*=0.85; Table 2).

### Delays in Care and 1-Year Outcomes for Minority Populations

In the overall cohort, Hispanic ethnicity was associated with a longer interval from presentation to diagnostic confirmation after adjustment for DS-MMS versus MMS status (ratio of days, 1.28 [95% CI, 1.08–1.51]; *P*=0.01; Table 4). In contrast, non-White race and Medicare/Medicaid insurance were not significantly associated with stroke at presentation, acute aspirin utilization, delays in care, mRS score ≥2 at 1 year, epilepsy at 1 year, or spasticity at 1 year. In the DS-MMS–only analysis, Hispanic ethnicity showed a stronger association with delayed diagnostic confirmation (ratio of days, 1.86 [95% CI, 1.27–2.72]; *P*=0.002), whereas the other race, ethnicity, and insurance associations were not statistically significant. In unadjusted ordinal logistic regression, longer delay was associated with worse mRS score at 1 year (OR, 1.44 [95% CI, 1.06–1.94]; *P*=0.02); however, after adjustment for DS status, delay was no longer independently associated with worse mRS score (aOR, 1.25 [95% CI, 0.90–1.73]; *P*=0.19) or spasticity (aOR, 0.74 [95% CI, 0.50–1.10]; *P*=0.13).

**Table 4.**
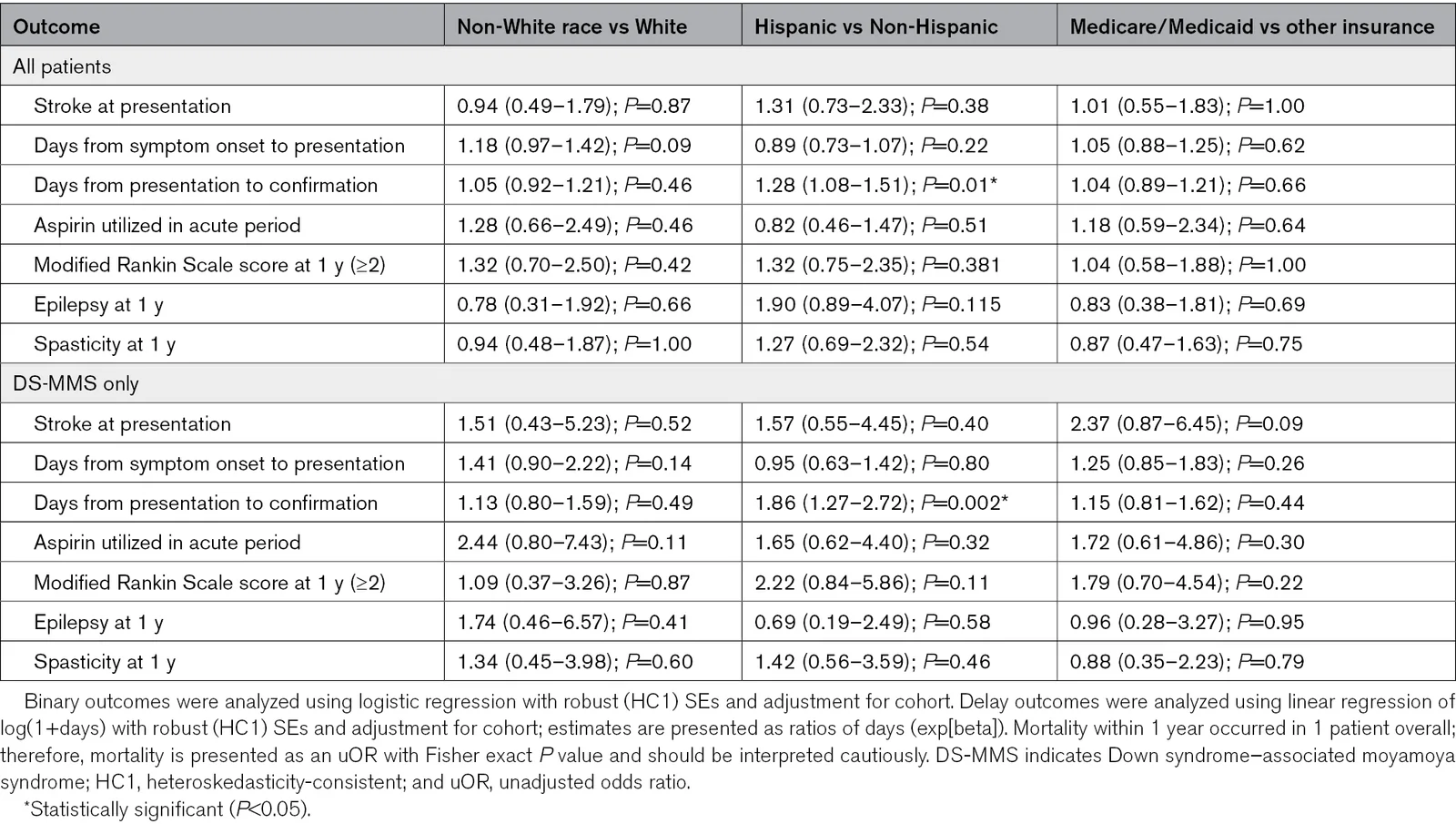
Associations of Race and Ethnicity and Insurance Status With Delays in Care and 1-Year Outcomes

## Discussion

Children with DS represent a distinct high-risk phenotype of moyamoya, characterized by delayed recognition, atypical early symptom attribution, and a markedly increased likelihood of stroke at initial presentation. Although delays were observed, our data suggest that disease biology and recognition failure, rather than delay alone, drive this stroke-predominant presentation. The DS-MMS cohort was >9× more likely to present with stroke and significantly more likely to experience greater 1-year neurological morbidity. Although race and insurance status were not associated with diagnostic delays, Hispanic ethnicity was independently associated with a 1.28-fold longer interval from clinical presentation to diagnostic confirmation. This suggests that once children with DS enter the medical system, those of Hispanic ethnicity may face unique systemic handoff barriers.

A central finding was that children with DS-MMS experienced delays at 2 clinically meaningful stages: the interval from symptom onset to presentation and the interval from presentation to diagnostic confirmation. Importantly, these delays were not minor; the DS-MMS group’s delays were meaningfully longer and remained so after multivariable adjustment. While prior studies in MMS have reported misdiagnosis,^13^ delays in primary evaluation have not been explored. In pediatric stroke and cerebrovascular disease, delays are consequential because they reduce the opportunity to identify transient ischemic symptoms; however, our findings suggest that underlying disease vulnerability and recognition failure may play a more dominant role in stroke-predominant presentation in DS-MMS.^14^ From a systems perspective, the presence of delays both before and after initial presentation suggests that DS-MMS is vulnerable to failure at multiple points in the diagnostic pathway, including symptom recognition, triage, and escalation to vascular imaging.

Several plausible mechanisms may contribute to these delays. First, early symptoms of MMS, particularly transient weakness, episodic speech disturbance, headaches, behavioral changes, or subtle gait alterations, may be less readily recognized as cerebrovascular red flags in children with DS because of baseline neurodevelopmental differences, communication challenges, or co-occurring conditions. This diagnostic overshadowing is well described across pediatric care for children with complex neurodevelopmental profiles and may lead to under-recognition of evolving focal neurological symptoms until a more overt event occurs.^15,16^ Our data strongly support the presence of diagnostic overshadowing, particularly as it relates to baseline intellectual and communication profiles. We found that patients with severe ID experienced a greater delay in time to presentation and diagnosis than in those with mild impairment. Furthermore, we observed a paradoxical relationship with communication status: verbal children experienced significantly longer delays to both presentation and diagnostic confirmation than their nonverbal peers. This may reflect the nature of their symptoms; nonverbal children frequently presented with sudden, undeniable focal deficits (eg, overt stroke in over 80% of our nonverbal cohort), triggering immediate emergency evaluation. In contrast, verbal children who could articulate auras, headaches, or transient sensory symptoms may have their early warning signs incorrectly attributed to behavioral changes or benign etiologies, delaying neurovascular imaging until deficits become persistent. Second, children with DS frequently interface with multiple subspecialties, and new symptoms may be initially attributed to more common DS-associated conditions (eg, sleep-disordered breathing, thyroid disease, or behavioral/developmental fluctuations) rather than prompting vascular neuroimaging. Third, the pathway from first presentation to confirmation may require coordination across emergency care, neurology, radiology, and neurosurgery; in resource-constrained settings or in the presence of communication barriers, these pathways may be slower and warrant additional study.^17,18^ These interpretations highlight a practical implication for pediatric practice: improving outcomes in DS-MMS may depend as much on earlier recognition and streamlined evaluation pathways as on the surgical or medical interventions that follow diagnosis. Future work should identify the most common sentinel symptoms in children with DS that precede stroke presentation and validate a standardized triage pathway for expedited imaging and referral.

Consistent with the observed delays in diagnosis, the authors observed differences in presentation phenotype in that children with DS were substantially more likely to present with stroke as the primary presenting symptom rather than nonstroke symptoms. This difference remained large even after adjustment for demographic and access-related factors. Clinically, this pattern suggests that children with DS may be reaching medical attention later in the course of the disease or after an event that produces persistent deficits, rather than being identified during a window of transient symptoms. One plausible explanation is that transient or subjective symptoms, including headache, sensory changes, dizziness, or brief focal weakness, may be more difficult for children with baseline ID or communication differences to recognize, describe, or localize. As a result, early transient ischemic attack–like symptoms may be underreported, misattributed to behavioral or developmental variability, or recognized only once symptoms become persistent or objectively observable. This hypothesis is consistent with our data in that children with DS-MMS were more likely to have posterior circulation involvement as well, which may reflect either more advanced disease at presentation or differential disease biology in DS-MMS.

As MMS can be intervened on surgically, the importance of early identification remains high. The observed rise in systolic BP percentiles preceding diagnosis suggests a potential early physiological signal of evolving cerebrovascular compromise. This study demonstrated a progressive rise in BP in individuals with DS-MMS over a 2-year period before presentation. These individuals started below MMS cases, consistent with previously published literature on lower BP profiles in individuals with DS^19^ but subsequently surpassed the MMS cohort 6 months before presentation. Although BP elevation remains a pragmatic early warning marker of cerebrovascular vulnerability in DS, its lack of specificity limits its use as a standalone screening tool but supports its role as part of a multimodal risk stratification approach.^9^ Combining BP changes with noninvasive diagnostics, such as transcranial Doppler ultrasound, represents a pragmatic and clinically feasible strategy for early detection that warrants prospective validation.^20–23^ These findings support a lower threshold for expedited vascular imaging in children with DS who develop new neurological symptoms, particularly in the context of rising BP percentiles, as part of a targeted early detection strategy. Earlier identification of MMS in individuals with DS may reduce stroke presentation and improve long-term neurological outcomes. Prospective validation of screening strategies incorporating clinical symptoms, BP trajectories, and noninvasive vascular imaging is warranted.

This study has several strengths. We used prespecified clinical hypotheses grounded in meaningful patient-centered outcomes, evaluated both presentation phenotype and 1-year morbidity, and applied multivariable models aligned with clinically relevant confounders. At the same time, several limitations warrant consideration. The study design is observational and retrospective, which introduces the potential for selection bias and residual confounding. The DS cohort was derived from a prospectively curated database, whereas the comparison cohort was identified via *International Classification of Diseases* coding, introducing potential differences in case ascertainment that may bias toward more clinically recognized DS-MMS cases. Within these data, severity bias is also likely to be present given the data were derived from 5 quaternary academic medical centers. Further, preventative screening measures and availability of imaging modalities changed dramatically during the study period (1998–2025), particularly for individuals with sickle cell disease,^24,25^ which may have influenced the likelihood of delays captured. However, the primary associations between DS status and diagnostic delays were robust in sensitivity analyses incorporating era of diagnosis. Missingness and variable completion resulted in variable denominators across outcomes, and rare events (eg, mortality) limited stable estimation. Some variables (including BP elevation and certain outcome categories) were captured categorically and were subject to variability in clinical documentation and abstraction, which may reduce sensitivity to clinically important gradients of risk. Although neurodevelopmental status was associated with delays within the DS-MMS cohort, similar analyses could not be reliably performed in the MMS cohort because ID and communication impairment were uncommon and sparsely distributed. As a result, it was not possible to determine whether the relationship between neurodevelopmental status and delay generalizes beyond DS-MMS. Furthermore, preexisting neurodevelopmental disability may have influenced 1-year functional outcomes, particularly mRS score and cognitive outcomes. Although baseline ID and communication status were captured descriptively, a standardized prestroke mRS score or comparable functional measure was not available and therefore could not be included as a covariate in the primary outcome models. Finally, the data reflect the characteristics of the participating sites and referral patterns, and generalizability to other settings may be limited.

## Conclusions

In summary, the authors found that children with DS-MMS experience longer diagnostic delays and are more likely to present with stroke than individuals with MMS without DS. Although unadjusted analyses suggested an association between delay and worse outcomes, this relationship was attenuated after accounting for DS status, suggesting that underlying disease vulnerability may be the dominant driver of morbidity. Prospective multicenter studies are needed to validate DS-specific sentinel symptoms, define risk stratification tools incorporating BP trajectories and noninvasive imaging, and determine whether earlier identification reduces stroke presentation and improves long-term outcomes.

## Article Information

### Disclosures

Dr Mayne reports grants from the American Heart Association. Dr Skotko reports compensation from New Zealand Down Syndrome Association for other services; compensation from American College of Osteopathic Obstetricians and Gynecologists for other services; compensation from Mano Institute for other services; compensation from Hippocrates Academy for other services; compensation from Case Western Reserve University for consultant services; grants from Boston College; other intellectual property for book royalties; compensation from 21 Club for other services; grants from Dartmouth Hitchcock Medical Center; compensation from South Shore Hospital for other services; compensation from Gerson Lehrman Group for consultant services; compensation from Boston University Medical Center for other services; compensation from Health Advances, LLC for expert witness services; compensation from Georgia Down Syndrome Association for other services; compensation from Goodell DeVries Law Firm for expert witness services; compensation from Guidepoint Global for consultant services; grants from AC Immune; grants from F. Hoffmann-La Roche; compensation from University of Massachusetts for expert witness services; compensation from Bergen Law Firm for expert witness services; compensation from Gerson Lehrman Group for consultant services; compensation from Acta Pharmaceutical for consultant services; grants from LuMind IDSC Down Syndrome Foundation; compensation from Swedish Down syndrome association for other services; compensation from Greenberg Traurig for expert witness services; compensation from Down Syndrome Advocacy Foundation for other services; compensation from Down Syndrome Association of NSW Australia for other services; and compensation from Las Vegas Down Syndrome Connections for other services. Dr Rafii reports grants from EISAI INC; compensation from Aerska for consultant services; compensation from Alzheon for data and safety monitoring services; compensation from AC Immune for consultant services; compensation from Positrigo for consultant services; compensation from Sumitomo Dainippon Pharma Co, Ltd for consultant services; compensation from Alnylam Pharmaceuticals Inc for consultant services; grants from Eli Lilly and Company; and compensation from Biohaven Pharmaceuticals Inc for data and safety monitoring services. The other authors report no conflicts.

### Supplemental Material

Supplemental Methods

Tables S1–S2

STROBE Checklist

## Supplementary Material


