# Supplementary material for: Delayed Recognition and Stroke-Predominant Presentation in Down Syndrome–Associated Moyamoya Syndrome
Source: Stroke. 2026 Jul 24;57(9):2731–43. doi: 10.1161/STROKEAHA.126.056969 (PMC13439558; doi:10.1161/STROKEAHA.126.056969)
Supplement: Supplementary file 1 [file str-57-2731-s001.pdf]

Methods S1: Definitions for sub-optimally treated, untreated, clinically significant disease and insignificant disease for patients previously diagnosed with hypothyroidism, heart disease or obstructive sleep apnea.

1. Hypothyroidism (defined either by previous ICD-9 or ICD-10 code, treatment with levothyroxine, or serum thyrotropin >4.5)
    a. Suboptimal treatment is defined as ≥2 changes to any medication in the past 2 years used to treat hypothyroidism or the use of a second (or more) agent for the control of hypothyroidism.
    b. Untreated is defined as patients with a previous diagnosis of hypothyroidism who have not received any pharmacotherapy for ≤1 year.
    c. Clinically significant disease is defined as patients experiencing any clinical comorbidity that could be linked to hypothyroidism after laboratory diagnosis of hypothyroidism including but not limited to the following: fatigue, memory issues (excluding developmental delay given patient population), excessive weight gain, chronic constipation, active or history of pleural effusions, hair loss, myxedema, bradycardia (persistent), menstrual irregularities, or thyroid crisis.
        i. All patients with a history of autoimmune thyroid disease were excluded.
    d. Clinically insignificant disease is defined as any patient with a previous diagnosis of hypothyroidism as above treated on either no pharmacotherapy or monotherapy with either normalization of laboratory values or lack of any comorbid symptoms as defined above. No >1 alteration to drug monotherapy was permitted in the past year in this group.
2. Heart disease is defined as any cardiac condition causing current or previous abnormalities in cardiac function, included both congenital and acquired disease.
    a. Suboptimal treatment is defined as clinically symptomatic but without deficits in ability to perform daily activities. Symptomatic features included hypoxemia, syncope, swelling, edema, chronic pulmonary disease (including pulmonary hypertension), fatigability, or orthopnea.
    b. Untreated disease is defined as any patient with cardiac dysfunction that required any previous pharmacotherapy (but not current) or with clinically symptomatic disease that causes disability or reduced ability to perform daily activities.
    c. Clinically significant disease is defined as any patient with cardiac dysfunction that requires any pharmacotherapy or with clinically symptomatic disease that causes disability or reduced ability to perform daily activities.
    d. Clinically insignificant disease is defined as any patient with no cardiac dysfunction or clinically symptomatic disease as defined above in the past 2 years. Patients in this group were required to have an echocardiogram in the past 3 years demonstrating normal cardiac function for their age.
3. OSA is defined as previous ICD-9 or ICD-10 code diagnosis, sleep study with an apnea–hypopnea index ≥5.0, or clinical history of snoring with witnessed apnea.

a. Suboptimal treatment is defined as any patient requiring positive pressure ventilation with subsequent normalization of apnea–hypopnea index to <5.0 but with >2 adjustments of positive pressure ventilation settings in the past 2 years.
b. Untreated disease is defined as a diagnosis of OSA as above but without any clinical intervention made.
c. Clinically significant disease is defined as any patient receiving positive pressure ventilation without correction of apnea–hypopnea index to <5.0 or patients with a diagnosis of OSA and any comorbid symptomatic condition including but not limited to the following: excessive daytime sleepiness, fatigue, need for daytime napping, irritability, attention issues (including diagnosis of attention-deficit/hyperactivity disorder), impulsivity or aggression at school (for patients <18 years old), headache, chronic hypertension, or memory issues.
d. Clinically insignificant disease is defined as patients with a previous diagnosis of OSA as above with normalization of apnea–hypopnea index to <5.0 and with ≤1 adjustment of positive pressure ventilation in the past 2 years. Patients should have no comorbid symptomatic conditions as defined above.

Table S1. Clinical and demographic data for individuals with Down syndrome and moyamoya (DS-MMS) versus moyamoya without Down syndrome (MMS) with incomplete data resulting in exclusion from analysis.

**Variable**	**DS-MMS** (n = 12)	**MMS** (n = 48)	**All** (n = 60)	**p Value**
Demographics and Baseline Clinical Data				
Sex				0.31
Male	8 (66.7%)	21 (43.7%)	29 (48.3%)	
Female	4 (33.3%)	24 (50.0%)	28 (46.7%)	
Unknown	0 (0%)	3 (6.3%)	3 (5.0%)	
Race				0.22
White	7 (58.3%)	33 (68.8%)	40 (66.7%)	
Black/African American	2 (16.7%)	3 (6.3%)	5 (8.3%)	
Asian	2 (16.7%)	3 (6.3%)	5 (8.3%)	
Other	1 (8.3%)	1 (2.1%)	2 (3.3%)	
Unknown	0 (0%)	8 (16.7%)	8 (13.3%)	
Hispanic Ethnicity				0.43
Yes	4 (33.3%)	8 (16.7%)	12 (20.0%)	
No	4 (33.3%)	21 (43.7%)	25 (41.7%)	
Unknown	4 (33.3%)	19 (39.5%)	23 (38.3%)	
Primary Language at Home				0.47
English	5 (41.7%)	12 (25.0%)	17 (28.3%)	
Spanish	1 (8.3%)	3 (6.3%)	4 (6.7%)	
Unknown	6 (50.0%)	33 (68.8%)	39 (65.0%)	
Insurance Type				0.82
Medicare/Medicaid	2 (16.7%)	5 (10.4%)	7 (11.7%)	
Commercial	2 (16.7%)	7 (14.6%)	9 (15.0%)	
Other	1 (8.3%)	2 (4.2%)	3 (5.0%)	
Unknown	7 (58.3%)	34 (70.8%)	41 (68.3%)	
Primary MMS Risk				**<0.001**
Down Syndrome	12 (100%)	0 (0%)	12 (20.0%)	
Neurofibromatosis Type I	0 (0%)	3 (6.3%)	3 (5.0%)	
Sickle Cell Disease	0 (0%)	9 (18.8%)	9 (15.0%)	

Prior Radiotherapy	0 (0%)	7 (14.6%)	7 (11.7%)	
Progeria	0 (0%)	1 (2.1%)	1 (1.7%)	
None/Cryptogenic	0 (0%)	7 (14.6%)	7 (11.7%)	
Unknown	0 (0%)	21 (43.8%)	21 (35.0%)	
Congenital Heart Disease				**<0.001**
Yes	4 (33.3%)	1 (2.1%)	5 (8.3%)	
No	3 (25.0%)	5 (10.4%)	8 (13.3%)	
Unknown	5 (41.7%)	42 (87.5%)	47 (78.3%)	
Hypothyroidism				0.69
Yes	2 (16.7%)	4 (8.3%)	6 (10.0%)	
No	2 (16.7%)	9 (18.8%)	11 (18.3%)	
Unknown	8 (66.7%)	35 (72.9%)	43 (71.7%)	
Autoimmune Disease				0.62
Yes	0 (0%)	1 (2.1%)	1 (1.7%)	
No	2 (16.7%)	4 (8.3%)	6 (10.0%)	
Unknown	10 (83.3%)	43 (89.6%)	53 (88.3%)	
Obstructive Sleep Apnea				**0.002**
Yes	4 (33.3%)	2 (4.2%)	6 (10.0%)	
No	2 (16.7%)	2 (4.2%)	4 (6.7%)	
Unknown	6 (50.0%)	44 (91.7%)	50 (83.3%)	
Hematologic Abnormalities[α]				0.92
Yes	1 (8.3%)	6 (12.5%)	7 (11.7%)	
No	1 (8.3%)	4 (8.3%)	5 (8.3%)	
Unknown	10 (83.3%)	38 (79.2%)	48 (80.0%)	
Baseline Intellectual Disability				0.42
None	0 (0%)	10 (20.8%)	10 (16.7%)	
Mild	2 (16.7%)	6 (12.5%)	8 (13.3%)	
Moderate	1 (8.3%)	3 (6.3%)	4 (6.7%)	
Severe	1 (8.3%)	1 (2.1%)	2 (3.3%)	
Unknown	8 (66.7%)	28 (50.0%)	36 (60.0%)	
Communication Status				0.13
Verbal	1 (8.3%)	4 (8.3%)	5 (8.3%)	
Minimally Verbal and Non-Verbal	1 (8.3%)	0 (0%)	1 (1.7%)	

Unknown	10 (83.3%)	44 (91.7%)	54 (90.0%)	

Legend: α: defined as non-genetic hematologic abnormalities such as anemia or thrombocytosis. IQR = interquartile range.

Table S2: Clinical and demographic data for individuals with Down syndrome and moyamoya (DS-MMS) versus moyamoya without Down syndrome (MMS) excluded from analysis due to clinically significant, uncontrolled or sub-optimally treated medical problems.

**Variable**	**DS-MMS** (n = 10)	**MMS** (n = 3)	**All** (n = 13)	**p Value**	**95% CI**
Exclusion Rationale					
Primary Exclusion Reason				0.26	**-**
Hypothyroidism	4 (40.0%)	0 (0%)	4 (30.8%)		
Clinically Significant	1/4 (25.0%)	0 (0%)	1/4 (25.0%)		
Uncontrolled	1/4 (25.0%)	0 (0%)	1/4 (25.0%)		
Sub-optimal Treatment	2/4 (50.0%)	0 (0%)	2/4 (50.0%)		
Cardiac Disease	4 (40.0%)	2 (66.7%)	6 (46.2%)		
Clinically Significant	3/4 (75.0%)	1/2 (50.0%)	4/6 (66.7%)		
Uncontrolled	1/4 (25.0%)	1/2 (50.0%)	2/6 (33%)		
Sub-optimal Treatment	0/4 (0%)	0/2 (0%)	0/6 (0%)		
Obstructive Sleep Apnea	2 (20.0%)	1 (33.3%)	3 (23.1%)		
Clinically Significant	0/2 (0%)	0/1 (0%)	0/3 (0%)		
Uncontrolled	2/2 (100%)	1/1 (100%)	3/3 (100%)		
Sub-optimal Treatment	0/2 (0%)	0/1 (0%)	0/3 (0%)		
Demographics and Baseline Clinical Data					
Sex				1.00	-36.3-54.5
Male	5 (50.0%)	1 (33.3%)	6 (46.2%)		
Female	5 (50.0%)	2 (66.7%)	7 (53.8%)		
Race				0.70	-
White	7 (70.0%)	2 (66.7%)	9 (69.2%)		
Black/African American	1 (10.0%)	0 (0%)	1 (7.7%)		
Asian	1 (10.0%)	1 (33.3%)	2 (15.4%)		
Other	1 (10.0%)	0 (0%)	1 (7.7%)		
Hispanic Ethnicity	4 (40.0%)	1 (33.3%)	5 (38.5%)	1.00	-44.8-46.2
Primary Language at Home				0.79	-
English	7 (70.0%)	2 (66.7%)	9 (69.2%)		
Spanish	2 (20.0%)	1 (33.3%)	3 (23.1%)		
Other	1 (10.0%)	0 (0%)	1 (7.7%)		
Insurance Type				0.85	-

Medicare/Medicaid	6 (60.0%)	2 (66.7%)	8 (61.5%)		
Commercial	3 (30.0%)	1 (33.3%)	4 (30.8%)		
Other	1 (10.0%)	0 (0%)	1 (7.7%)		
Primary MMS Risk				**<0.001**	-
Down Syndrome	10 (100%)	0 (0%)	10 (76.9%)		
Neurofibromatosis Type I	0 (0%)	1 (33.3%)	1 (7.7%)		
Sickle Cell Disease	0 (0%)	1 (33.3%)	1 (7.7%)		
Prior Radiotherapy	0 (0%)	0 (0%)	0 (0%)		
Progeria	0 (0%)	0 (0%)	0 (0%)		
None/Cryptogenic	0 (0%)	1 (33.3%)	1 (7.7%)		
Congenital Heart Disease	4 (40.0%)	2 (66.7%)	6 (46.2%)	0.56	-62.4-27.5
Hypothyroidism	4 (40.0%)	0 (0%)	4 (30.8%)	0.50	-20.7-68.7
Autoimmune Disease	2 (20.0%)	0 (0%)	2 (15.4%)	1.00	-37.9-51.0
Obstructive Sleep Apnea	2 (20.0%)	1 (33.3%)	3 (23.1%)	1.00	-61.4-27.9
Hematologic Abnormalities[α]	1 (10.0%)	0 (0%)	1 (7.7%)	1.00	-46.7-40.4
Baseline Intellectual Disability				0.23	-
None	0 (0%)	2 (66.7%)	2 (15.4%)		
Mild	3 (30.0%)	1 (33.3%)	4 (30.8%)		
Moderate	4 (40.0%)	0 (0%)	4 (30.8%)		
Severe	3 (30.0%)	0 (0%)	3 (23.1%)		
Communication Status				0.56	-62.4-27.5
Verbal	4 (40.0%)	2 (66.7%)	6 (46.2%)		
Minimally Verbal and Non-Verbal	6 (60.0%)	1 (33.3%)	7 (53.8%)		
Moyamoya Clinical Features					
Age at Presentation (median, IQR)	7.8 (6.0-12.5)	7.1 (5.8-8.9)	7.5 (5.9-10.8)	0.37	-3.2-4.6
Era of Diagnosis				0.85	-
1998-2006	2 (20.0%)	1 (33.3%)	3 (23.1%)		
2006-2014	3 (30.0%)	1 (33.3%)	4 (30.8%)		
2015-2025	5 (50.0%)	1 (33.3%)	6 (46.2%)		
Presenting Symptoms				0.24	-
Stroke	7 (70.0%)	1 (33.3%)	8 (61.5%)		
Transient Ischemic Attack (TIA)	1 (10.0%)	1 (33.3%)	2 (15.4%)		
Headache	1 (10.0%)	1 (33.3%)	2 (15.4%)		

Language Deficit	0 (0%)	0 (0%)	0 (0%)		
Cognitive Decline	0 (0%)	0 (0%)	0 (0%)		
Abnormal Movements	0 (0%)	0 (0%)	0 (0%)		
Other	1 (10.0%)	0 (0%)	1 (7.7%)		
Stroke/TIA Symptoms at Presentation	8 (80.0%)	2 (66.7%)	10 (76.9%)	0.59	-
Hemiparesis	3/8 (37.5%)	1/2 (50.0%)	4/10 (40.0%)		
Focal Weakness (Single Extremity)	2/8 (25.0%)	1/2 (50.0%)	3/10 (30.0%)		
Sensory Deficit	0/8 (0%)	0/2 (0%)	0/10 (0%)		
Dysarthria	1/8 (12.5%)	0/2 (0%)	1/10 (10.0%)		
Ocular/Visual Symptoms	0/8 (0%)	0/2 (0%)	0/10 (0%)		
Bulbar/Facial Weakness	1/8 (12.5%)	0/2 (0%)	1/10 (10.0%)		
Visual Field Cut	0/8 (0%)	0/2 (0%)	0/10 (0%)		
Gait Abnormality	1/8 (12.5%)	0/2 (0%)	1/10 (10.0%)		
Days Between Symptom Onset and Presentation (median, IQR)	31 (17-65)	18 (14-24)	24 (16-54)	0.29	-8-52
Days Before Confirmation of Diagnosis (median, IQR)	3 (2-7)	2 (1-3)	3 (1-6)	0.17	-1-5
Radiographic Stroke at Presentation	7 (70.0%)	2 (66.7%)	9 (69.2%)	0.76	-
Left Only	0/7 (0%)	0/2 (0%)	0 (0%)		
Right Only	1/7 (14.3%)	0/2 (0%)	1 (11.1%)		
Bilateral	6/7 (85.7%)	2/2 (100%)	8 (88.9%)		
Bilateral MMS at Presentation	8 (80.0%)	2 (66.7%)	10 (76.9%)	1.00	-27.9-61.4
Laterality of Vascular Disease				0.76	-
Unilateral Left Sided	1 (10.0%)	0 (0%)	1 (7.7%)		
Unilateral Right Sided	1 (10.0%)	1 (33.3%)	2 (15.4%)		
Bilateral, Worse Disease on Left	4 (40.0%)	1 (33.3%)	5 (38.5%)		
Bilateral, Worse Disease on Right	4 (40.0%)	1 (33.3%)	5 (38.5%)		
Suzuki Grade (median, IQR)	3 (3-4)	3 (3-4)	3 (3-4)	0.78	-1-1
Posterior Involvement	3 (30.0%)	1 (33.3%)	4 (30.8%)	1.00	-53.1-37.4
Acute Interventions					
Acute Administration of Aspirin	3 (30.0%)	1 (33.3%)	4 (30.8%)	1.00	-53.1-37.4
Surgical Intervention				0.70	-
Direct Bypass	1 (10.0%)	0 (0%)	1 (7.7%)		
Indirect Bypass (EDAS, EDAMS, EMS)	8 (80.0%)	3 (100%)	11 (84.6%)		

No Surgical Intervention	1 (10.0%)	0 (0%)	1 (7.7%)		
Post-Operative Focal Deficits	1 (10.0%)	0 (0%)	1 (7.7%)	1.00	-46.7-40.4
Post-Operative Stroke	0 (0%)	0 (0%)	0 (0%)	1.00	-
Post-Operative Seizure	1 (10.0%)	0 (0%)	1 (7.7%)	1.00	-46.7-40.4
Post-Operative Hemorrhage	0 (0%)	0 (0%)	0 (0%)	1.00	-
Post-Operative Wound Infection	0 (0%)	0 (0%)	0 (0%)	1.00	-
Clinical Outcomes					
Modified Rankin Score (median, IQR)	2 (1-3)	1 (1-2)	2 (1-3)	0.21	-1-2
Mortality at one year	0 (0%)	0 (0%)	0 (0%)	1.00	-
Epilepsy at one year	2 (20.0%)	1 (33.3%)	3 (23.1%)	1.00	-61.4-27.9
Epilepsy and >2 anticonvulsants	1 (10.0%)	0 (0%)	1 (7.7%)	1.00	-46.7-40.4
Spasticity at one year	5 (50.0%)	1 (33.3%)	6 (46.2%)	1.00	-36.3-54.5
Need for spasticity medications at one year	4 (40.0%)	1 (33.3%)	5 (38.5%)	1.00	-44.8-46.2
Intellectual capabilities worse at one year	4 (40.0%)	1 (33.3%)	5 (38.5%)	1.00	-44.8-46.2
Stroke recurrence by one year	0 (0%)	0 (0%)	0 (0%)	1.00	-

Legend: α: defined as non-genetic hematologic abnormalities such as anemia or thrombocytosis. IQR = interquartile range.
